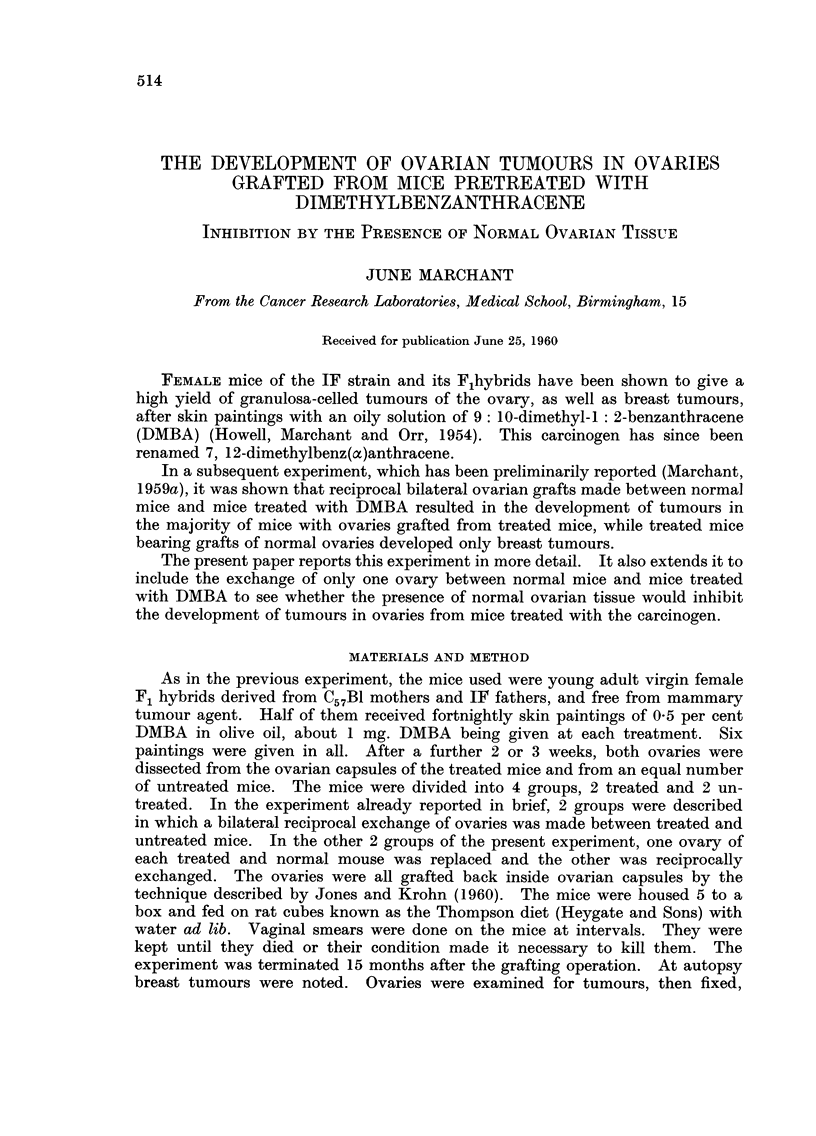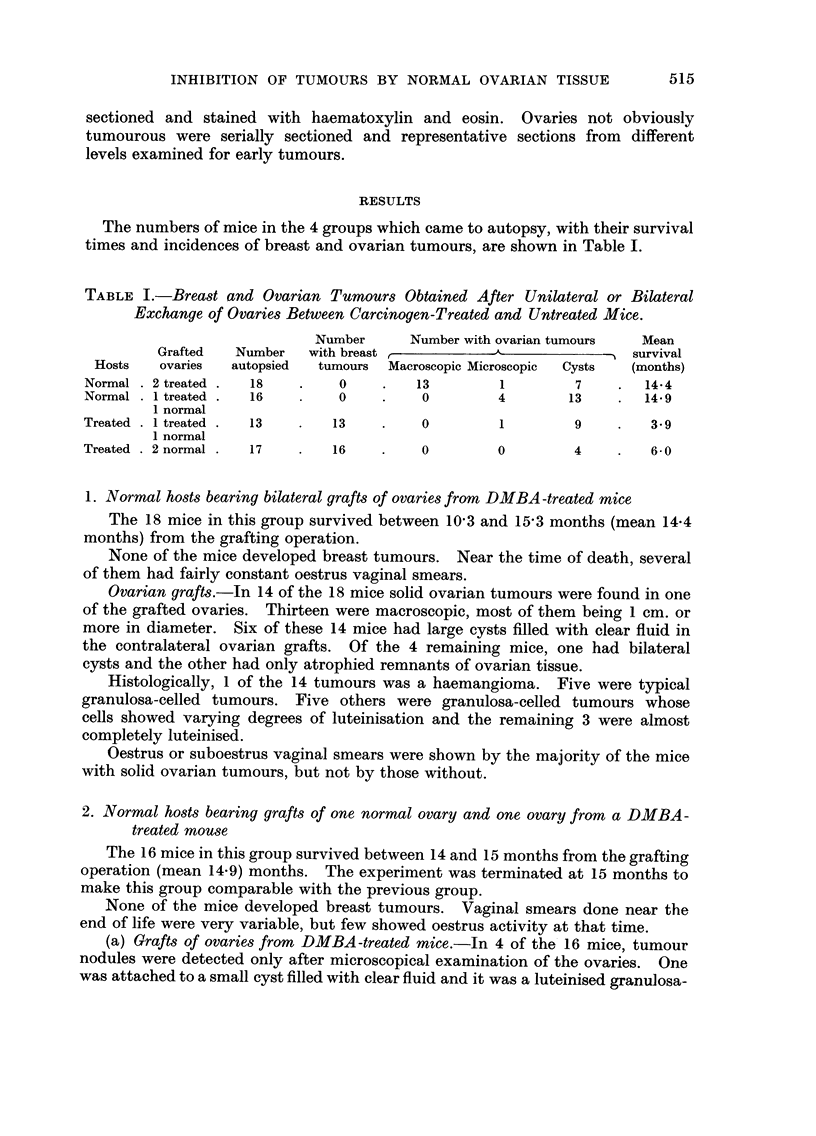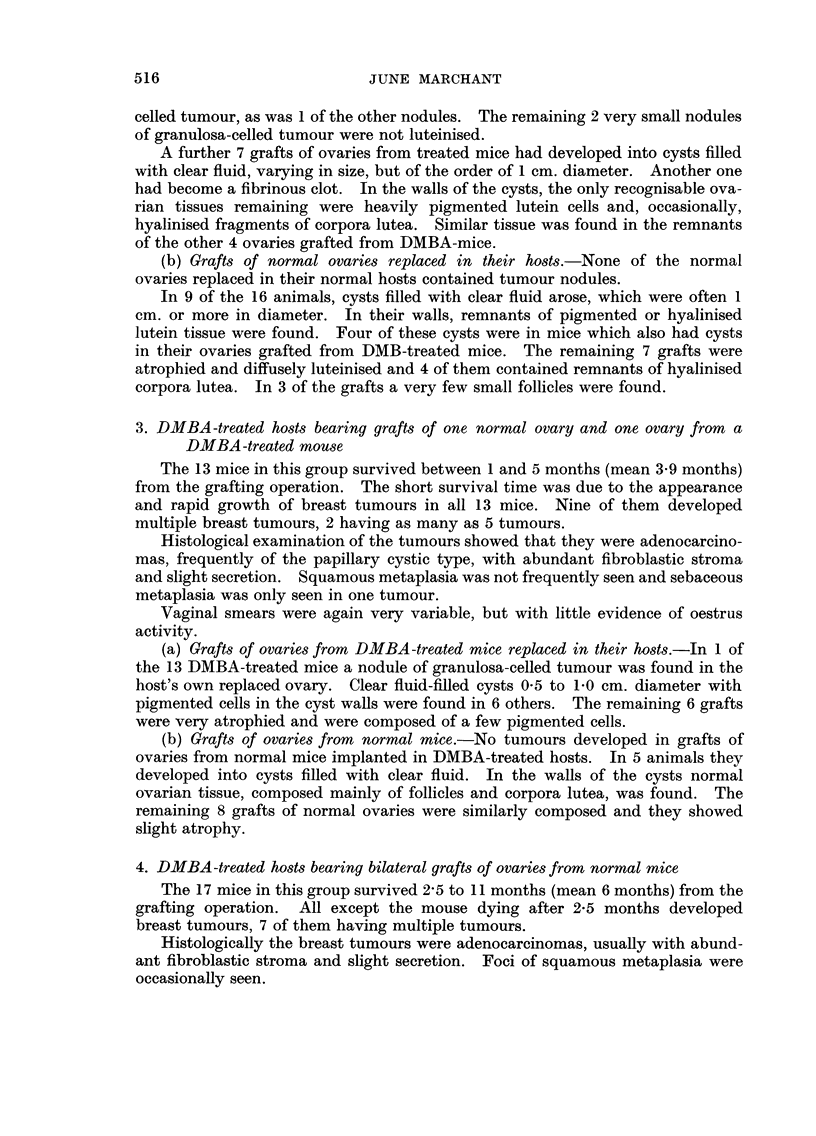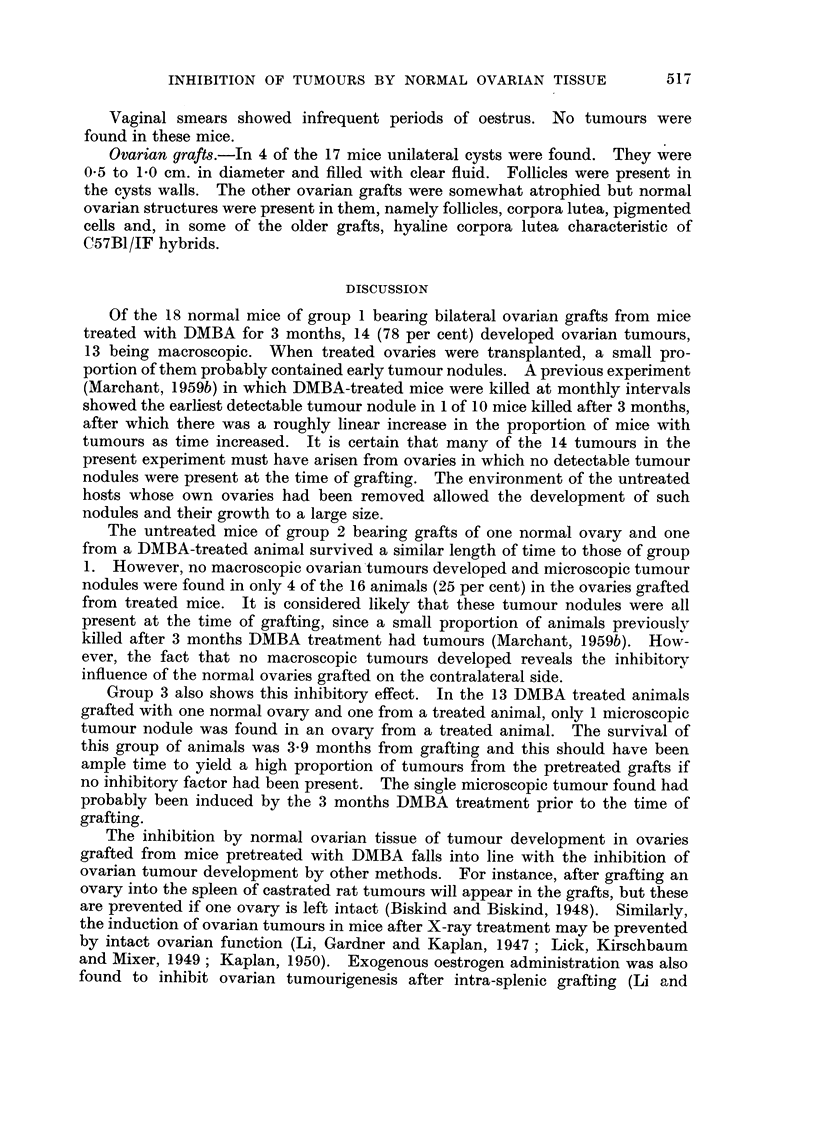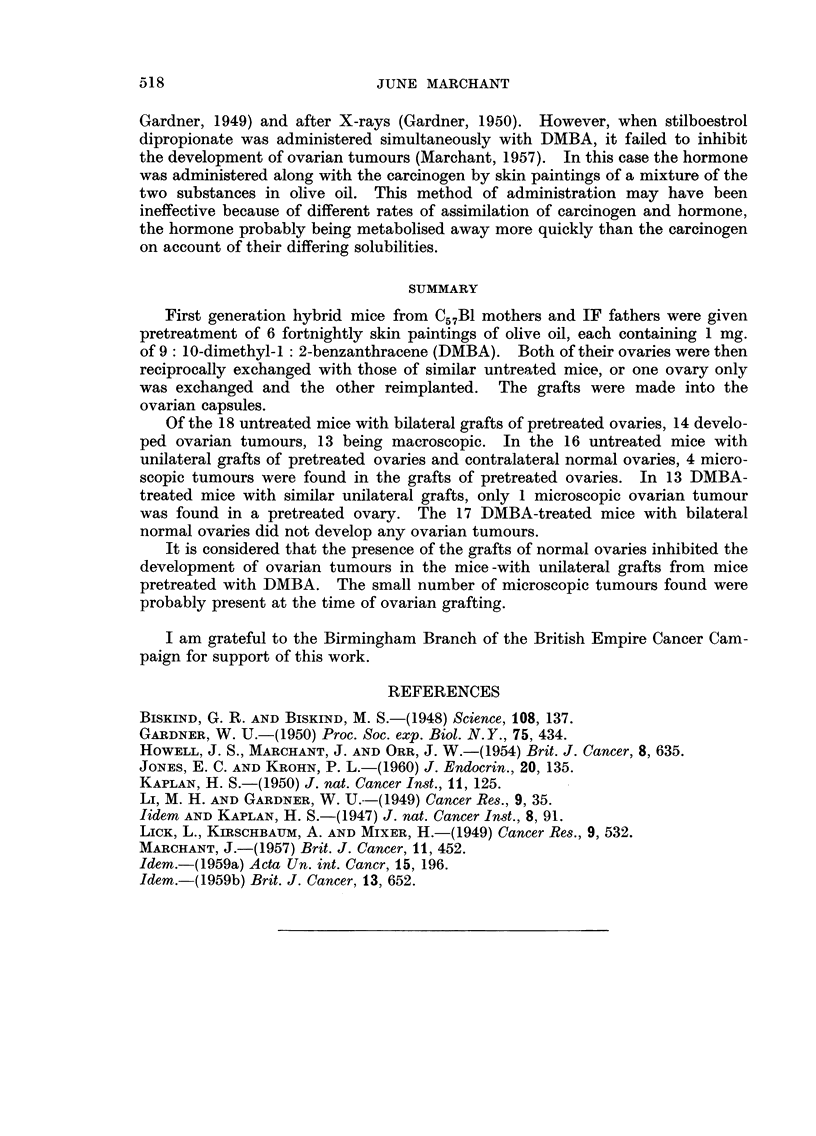# The Development of Ovarian Tumours in Ovaries Grafted from Mice Pretreated with Dimethylbenzanthracene

**DOI:** 10.1038/bjc.1960.57

**Published:** 1960-09

**Authors:** June Marchant


### THE DEVELOPMENT OF OVARIAN TUMOURS IN OVARIES GRAFTED FROM MICE PRETREATED WITH DIMETHYLBENZANTHRACENE

### INHIBITION BY THE PRESENCE OF NORMAL OVARIAN TISSUE

JUNE MARCHANT

*From the Cancer Research Laboratories, Medical School, Birmingham,* 15

Received for publication June 25, 1960

FEMALE mice of the IF strain and its $F_1$hybrids have been shown to give a high yield of granulosa-celled tumours of the ovary, as well as breast tumours, after skin paintings with an oily solution of 9 : 10-dimethyl-1 : 2-benzanthracene (DMBA) (Howell, Marchant and Orr, 1954). This carcinogen has since been renamed 7, 12-dimethylbenz($\alpha$)anthracene.

In a subsequent experiment, which has been preliminarily reported (Marchant, 1959*a*), it was shown that reciprocal bilateral ovarian grafts made between normal mice and mice treated with DMBA resulted in the development of tumours in the majority of mice with ovaries grafted from treated mice, while treated mice bearing grafts of normal ovaries developed only breast tumours.

The present paper reports this experiment in more detail. It also extends it to include the exchange of only one ovary between normal mice and mice treated with DMBA to see whether the presence of normal ovarian tissue would inhibit the development of tumours in ovaries from mice treated with the carcinogen.

### MATERIALS AND METHOD

As in the previous experiment, the mice used were young adult virgin female $F_1$ hybrids derived from $C_{57}Bl$ mothers and IF fathers, and free from mammary tumour agent. Half of them received fortnightly skin paintings of 0·5 per cent DMBA in olive oil, about 1 mg. DMBA being given at each treatment. Six paintings were given in all. After a further 2 or 3 weeks, both ovaries were dissected from the ovarian capsules of the treated mice and from an equal number of untreated mice. The mice were divided into 4 groups, 2 treated and 2 untreated. In the experiment already reported in brief, 2 groups were described in which a bilateral reciprocal exchange of ovaries was made between treated and untreated mice. In the other 2 groups of the present experiment, one ovary of each treated and normal mouse was replaced and the other was reciprocally exchanged. The ovaries were all grafted back inside ovarian capsules by the technique described by Jones and Krohn (1960). The mice were housed 5 to a box and fed on rat cubes known as the Thompson diet (Heygate and Sons) with water *ad lib.* Vaginal smears were done on the mice at intervals. They were kept until they died or their condition made it necessary to kill them. The experiment was terminated 15 months after the grafting operation. At autopsy breast tumours were noted. Ovaries were examined for tumours, then fixed, sectioned and stained with haematoxylin and eosin. Ovaries not obviously tumourous were serially sectioned and representative sections from different levels examined for early tumours.

### RESULTS

The numbers of mice in the 4 groups which came to autopsy, with their survival times and incidences of breast and ovarian tumours, are shown in Table I.

TABLE I.—*Breast and Ovarian Tumours Obtained After Unilateral or Bilateral Exchange of Ovaries Between Carcinogen-Treated and Untreated Mice.*

| Hosts | Grafted ovaries | Number autopsied | Number with breast tumours | Number with ovarian tumours: Macroscopic | Microscopic | Cysts | Mean survival (months) |
|---|---|---|---|---|---|---|---|
| Normal | 2 treated | 18 | 0 | 13 | 1 | 7 | 14·4 |
| Normal | 1 treated<br>1 normal | 16 | 0 | 0 | 4 | 13 | 14·9 |
| Treated | 1 treated<br>1 normal | 13 | 13 | 0 | 1 | 9 | 3·9 |
| Treated | 2 normal | 17 | 16 | 0 | 0 | 4 | 6·0 |

1. *Normal hosts bearing bilateral grafts of ovaries from DMBA-treated mice*

The 18 mice in this group survived between 10·3 and 15·3 months (mean 14·4 months) from the grafting operation.

None of the mice developed breast tumours. Near the time of death, several of them had fairly constant oestrus vaginal smears.

*Ovarian grafts.*—In 14 of the 18 mice solid ovarian tumours were found in one of the grafted ovaries. Thirteen were macroscopic, most of them being 1 cm. or more in diameter. Six of these 14 mice had large cysts filled with clear fluid in the contralateral ovarian grafts. Of the 4 remaining mice, one had bilateral cysts and the other had only atrophied remnants of ovarian tissue.

Histologically, 1 of the 14 tumours was a haemangioma. Five were typical granulosa-celled tumours. Five others were granulosa-celled tumours whose cells showed varying degrees of luteinisation and the remaining 3 were almost completely luteinised.

Oestrus or suboestrus vaginal smears were shown by the majority of the mice with solid ovarian tumours, but not by those without.

2. *Normal hosts bearing grafts of one normal ovary and one ovary from a DMBA-treated mouse*

The 16 mice in this group survived between 14 and 15 months from the grafting operation (mean 14·9) months. The experiment was terminated at 15 months to make this group comparable with the previous group.

None of the mice developed breast tumours. Vaginal smears done near the end of life were very variable, but few showed oestrus activity at that time.

(a) *Grafts of ovaries from DMBA-treated mice.*—In 4 of the 16 mice, tumour nodules were detected only after microscopical examination of the ovaries. One was attached to a small cyst filled with clear fluid and it was a luteinised granulosacelled tumour, as was 1 of the other nodules. The remaining 2 very small nodules of granulosa-celled tumour were not luteinised.

A further 7 grafts of ovaries from treated mice had developed into cysts filled with clear fluid, varying in size, but of the order of 1 cm. diameter. Another one had become a fibrinous clot. In the walls of the cysts, the only recognisable ovarian tissues remaining were heavily pigmented lutein cells and, occasionally, hyalinised fragments of corpora lutea. Similar tissue was found in the remnants of the other 4 ovaries grafted from DMBA-mice.

(b) *Grafts of normal ovaries replaced in their hosts.*—None of the normal ovaries replaced in their normal hosts contained tumour nodules.

In 9 of the 16 animals, cysts filled with clear fluid arose, which were often 1 cm. or more in diameter. In their walls, remnants of pigmented or hyalinised lutein tissue were found. Four of these cysts were in mice which also had cysts in their ovaries grafted from DMB-treated mice. The remaining 7 grafts were atrophied and diffusely luteinised and 4 of them contained remnants of hyalinised corpora lutea. In 3 of the grafts a very few small follicles were found.

### 3. *DMBA-treated hosts bearing grafts of one normal ovary and one ovary from a DMBA-treated mouse*

The 13 mice in this group survived between 1 and 5 months (mean 3·9 months) from the grafting operation. The short survival time was due to the appearance and rapid growth of breast tumours in all 13 mice. Nine of them developed multiple breast tumours, 2 having as many as 5 tumours.

Histological examination of the tumours showed that they were adenocarcinomas, frequently of the papillary cystic type, with abundant fibroblastic stroma and slight secretion. Squamous metaplasia was not frequently seen and sebaceous metaplasia was only seen in one tumour.

Vaginal smears were again very variable, but with little evidence of oestrus activity.

(a) *Grafts of ovaries from DMBA-treated mice replaced in their hosts.*—In 1 of the 13 DMBA-treated mice a nodule of granulosa-celled tumour was found in the host's own replaced ovary. Clear fluid-filled cysts 0·5 to 1·0 cm. diameter with pigmented cells in the cyst walls were found in 6 others. The remaining 6 grafts were very atrophied and were composed of a few pigmented cells.

(b) *Grafts of ovaries from normal mice.*—No tumours developed in grafts of ovaries from normal mice implanted in DMBA-treated hosts. In 5 animals they developed into cysts filled with clear fluid. In the walls of the cysts normal ovarian tissue, composed mainly of follicles and corpora lutea, was found. The remaining 8 grafts of normal ovaries were similarly composed and they showed slight atrophy.

### 4. *DMBA-treated hosts bearing bilateral grafts of ovaries from normal mice*

The 17 mice in this group survived 2·5 to 11 months (mean 6 months) from the grafting operation. All except the mouse dying after 2·5 months developed breast tumours, 7 of them having multiple tumours.

Histologically the breast tumours were adenocarcinomas, usually with abundant fibroblastic stroma and slight secretion. Foci of squamous metaplasia were occasionally seen.

Vaginal smears showed infrequent periods of oestrus. No tumours were found in these mice.

*Ovarian grafts.*—In 4 of the 17 mice unilateral cysts were found. They were 0·5 to 1·0 cm. in diameter and filled with clear fluid. Follicles were present in the cysts walls. The other ovarian grafts were somewhat atrophied but normal ovarian structures were present in them, namely follicles, corpora lutea, pigmented cells and, in some of the older grafts, hyaline corpora lutea characteristic of C57Bl/IF hybrids.

### DISCUSSION

Of the 18 normal mice of group 1 bearing bilateral ovarian grafts from mice treated with DMBA for 3 months, 14 (78 per cent) developed ovarian tumours, 13 being macroscopic. When treated ovaries were transplanted, a small proportion of them probably contained early tumour nodules. A previous experiment (Marchant, 1959*b*) in which DMBA-treated mice were killed at monthly intervals showed the earliest detectable tumour nodule in 1 of 10 mice killed after 3 months, after which there was a roughly linear increase in the proportion of mice with tumours as time increased. It is certain that many of the 14 tumours in the present experiment must have arisen from ovaries in which no detectable tumour nodules were present at the time of grafting. The environment of the untreated hosts whose own ovaries had been removed allowed the development of such nodules and their growth to a large size.

The untreated mice of group 2 bearing grafts of one normal ovary and one from a DMBA-treated animal survived a similar length of time to those of group 1. However, no macroscopic ovarian tumours developed and microscopic tumour nodules were found in only 4 of the 16 animals (25 per cent) in the ovaries grafted from treated mice. It is considered likely that these tumour nodules were all present at the time of grafting, since a small proportion of animals previously killed after 3 months DMBA treatment had tumours (Marchant, 1959*b*). However, the fact that no macroscopic tumours developed reveals the inhibitory influence of the normal ovaries grafted on the contralateral side.

Group 3 also shows this inhibitory effect. In the 13 DMBA treated animals grafted with one normal ovary and one from a treated animal, only 1 microscopic tumour nodule was found in an ovary from a treated animal. The survival of this group of animals was 3·9 months from grafting and this should have been ample time to yield a high proportion of tumours from the pretreated grafts if no inhibitory factor had been present. The single microscopic tumour found had probably been induced by the 3 months DMBA treatment prior to the time of grafting.

The inhibition by normal ovarian tissue of tumour development in ovaries grafted from mice pretreated with DMBA falls into line with the inhibition of ovarian tumour development by other methods. For instance, after grafting an ovary into the spleen of castrated rat tumours will appear in the grafts, but these are prevented if one ovary is left intact (Biskind and Biskind, 1948). Similarly, the induction of ovarian tumours in mice after X-ray treatment may be prevented by intact ovarian function (Li, Gardner and Kaplan, 1947 ; Lick, Kirschbaum and Mixer, 1949 ; Kaplan, 1950). Exogenous oestrogen administration was also found to inhibit ovarian tumourigenesis after intra-splenic grafting (Li and

Gardner, 1949) and after X-rays (Gardner, 1950). However, when stilboestrol dipropionate was administered simultaneously with DMBA, it failed to inhibit the development of ovarian tumours (Marchant, 1957). In this case the hormone was administered along with the carcinogen by skin paintings of a mixture of the two substances in olive oil. This method of administration may have been ineffective because of different rates of assimilation of carcinogen and hormone, the hormone probably being metabolised away more quickly than the carcinogen on account of their differing solubilities.

### SUMMARY

First generation hybrid mice from $C_{57}$Bl mothers and IF fathers were given pretreatment of 6 fortnightly skin paintings of olive oil, each containing 1 mg. of 9 : 10-dimethyl-1 : 2-benzanthracene (DMBA). Both of their ovaries were then reciprocally exchanged with those of similar untreated mice, or one ovary only was exchanged and the other reimplanted. The grafts were made into the ovarian capsules.

Of the 18 untreated mice with bilateral grafts of pretreated ovaries, 14 developed ovarian tumours, 13 being macroscopic. In the 16 untreated mice with unilateral grafts of pretreated ovaries and contralateral normal ovaries, 4 microscopic tumours were found in the grafts of pretreated ovaries. In 13 DMBA-treated mice with similar unilateral grafts, only 1 microscopic ovarian tumour was found in a pretreated ovary. The 17 DMBA-treated mice with bilateral normal ovaries did not develop any ovarian tumours.

It is considered that the presence of the grafts of normal ovaries inhibited the development of ovarian tumours in the mice-with unilateral grafts from mice pretreated with DMBA. The small number of microscopic tumours found were probably present at the time of ovarian grafting.

I am grateful to the Birmingham Branch of the British Empire Cancer Campaign for support of this work.

### REFERENCES

BISKIND, G. R. AND BISKIND, M. S.—(1948) *Science*, **108**, 137.
GARDNER, W. U.—(1950) *Proc. Soc. exp. Biol. N.Y.*, **75**, 434.
HOWELL, J. S., MARCHANT, J. AND ORR, J. W.—(1954) *Brit. J. Cancer*, **8**, 635.
JONES, E. C. AND KROHN, P. L.—(1960) *J. Endocrin.*, **20**, 135.
KAPLAN, H. S.—(1950) *J. nat. Cancer Inst.*, **11**, 125.
LI, M. H. AND GARDNER, W. U.—(1949) *Cancer Res.*, **9**, 35.
*Iidem* AND KAPLAN, H. S.—(1947) *J. nat. Cancer Inst.*, **8**, 91.
LICK, L., KIRSCHBAUM, A. AND MIXER, H.—(1949) *Cancer Res.*, **9**, 532.
MARCHANT, J.—(1957) *Brit. J. Cancer*, **11**, 452.
*Idem.*—(1959a) *Acta Un. int. Cancr*, **15**, 196.
*Idem.*—(1959b) *Brit. J. Cancer*, **13**, 652.